# Persistence of Aquatic Insects across Managed Landscapes: Effects of Landscape Permeability on Re-Colonization and Population Recovery

**DOI:** 10.1371/journal.pone.0054584

**Published:** 2013-01-24

**Authors:** Nika Galic, Geerten M. Hengeveld, Paul J. Van den Brink, Amelie Schmolke, Pernille Thorbek, Eric Bruns, Hans M. Baveco

**Affiliations:** 1 Department of Aquatic Ecology and Water Quality Management, Wageningen University, Wageningen, The Netherlands; 2 Alterra, Wageningen University and Research centre, Wageningen, The Netherlands; 3 Workgroup Movement Ecology, Netherlands Institute of Ecology, NIOO-KNAW, Wageningen, The Netherlands; 4 UFZ, Helmholtz Centre for Environmental Research, Department of Ecological Modelling, Leipzig, Germany; 5 Syngenta Ltd., Bracknell, United Kingdom; 6 Bayer CropScience, Monheim, Germany; Swansea University, United Kingdom

## Abstract

Human practices in managed landscapes may often adversely affect aquatic biota, such as aquatic insects. Dispersal is often the limiting factor for successful re-colonization and recovery of stressed habitats. Therefore, in this study, we evaluated the effects of landscape permeability, assuming a combination of riparian vegetation (edge permeability) and other vegetation (landscape matrix permeability), and distance between waterbodies on the colonization and recovery potential of weakly flying insects. For this purpose, we developed two models, a movement and a population model of the non-biting midge, *Chironomus riparius*, an aquatic insect with weak flying abilities. With the movement model we predicted the outcome of dispersal in a landscape with several linear water bodies (ditches) under different assumptions regarding landscape-dependent movement. Output from the movement model constituted the probabilities of encountering another ditch and of staying in the natal ditch or perishing in the landscape matrix, and was used in the second model. With this individual-based model of midge populations, we assessed the implications for population persistence and for recovery potential after an extreme stress event. We showed that a combination of landscape attributes from the movement model determines the fate of dispersing individuals and, once extrapolated to the population level, has a big impact on the persistence and recovery of populations. Population persistence benefited from low edge permeability as it reduced the dispersal mortality which was the main factor determining population persistence and viability. However, population recovery benefited from higher edge permeability, but this was conditional on the low effective distance that ensured fewer losses in the landscape matrix. We discuss these findings with respect to possible landscape management scenarios.

## Introduction

Human activities are changing aquatic ecosystems worldwide, by imposing multiple stressors to the aquatic compartment. This stress is imposed by e.g. physical alterations of the habitat, such as channelling of streams and rivers, chemical and nutrient runoff from agricultural practices, and introduction of invasive species [1]. These occurrences led to severe degradation of aquatic environments, with negative consequences for ecosystem services provided for human benefit [2], such as quality potable water, biological diversity and community structure resulting in aesthetic, cultural and recreational value. To counter this trend, many of such degraded ecosystems are currently undergoing comprehensive restoration projects, with a goal of recovering the native biota, and restoring a functioning ecosystem. Unfortunately, the success rate of such projects has been quite limited [3], [4]. One of the desired processes in aquatic restoration projects is the recovery of the native biota, through the colonization of the restored or stressed habitat, occurring almost exclusively via dispersal of individuals from nearby areas [3], [5], [6].

Species dispersal, therefore, has an especially vital role in ensuring population persistence across managed and disturbed landscapes. Dispersal is generally defined as moving away from the natal location or population, usually assuming crossing larger spatial scales, though the exact definition of dispersal has, however, often been left to the interpretation of different authors [7]. Colonization is found to be the limiting factor in many restoration efforts [3], [5], [8], as dispersing individuals might come across different barriers in the landscape that may limit their colonization success [3], [5], [9], [10]. Physical barriers include structures such as dams, bridges or roads [5]. Furthermore, landscape connectivity [11] and trophic constraints [12] may also limit colonization success.

Freshwater ecosystems in managed landscapes harbour a variety of invertebrate species, where aquatic insects are one of the major contributors to overall biomass production [13] and to the transfer of energy between the aquatic and terrestrial ecosystems [14]–[16].

Colonization and recovery of riverine insect populations typically follows the stream channel network, making, thus, the longitudinal connectivity essential [17]. However, colonization of more isolated riverine systems requires lateral dispersal, i.e. across landscapes and away from the aquatic habitat [8]. Many insect species are often weak flyers, carried by the wind, and seldom move laterally from their natal waterbody [18]–[20], though the evidence that this occurs more often than previously thought is increasing [13], [21]–[24]. Such species, including various chironomids [25]–[28], use riparian vegetation as windbreaks [29] and for the completion of their life-cycles. Riparian vegetation is, thus, beneficial for the protection and persistence of individual insects [30], [31], but can, at the same time, limit lateral dispersal of those individuals that would be, for instance, otherwise carried by the wind [25].

In this study we, therefore, evaluated the effects of landscape permeability, i.e. of riparian vegetation (edge permeability) and other vegetation (landscape matrix permeability), and distance between waterbodies on the colonization and recovery potential of weakly flying insects. We chose the non-biting midges, *Chironomus riparius*, as our model organisms, due to their importance in energy transfers in aquatic and terrestrial food webs [32], their global distribution and low flying capabilities. Chironomid dispersal usually includes three types of movement: initial movement after emergence to the resting site, swarming, i.e. mating behaviour and ovipositing flight of females [33]. In our model, we assume a single movement pattern to apply to all phases. To investigate the interplay between landscape permeability, distance between water bodies, and individual movement and the consequences for re-colonization and population recovery after a stress event, we developed two models. With a movement model we predicted the outcome of dispersal in a landscape with several linear water bodies (ditches) under different assumptions regarding landscape-dependent movement. The outcome of the movement model, in particular the individual probabilities of encountering another ditch (functional connectivity) and of staying in the natal ditch or perishing in the landscape matrix, was used in a population model. With this individual-based model we assessed the implications for population persistence (taking abundance as a proxy for viability) and in particular for recovery potential (i.e. time to recovery) after an extreme stress event.

## Results

### Movement Model Output

**Figure 1:**
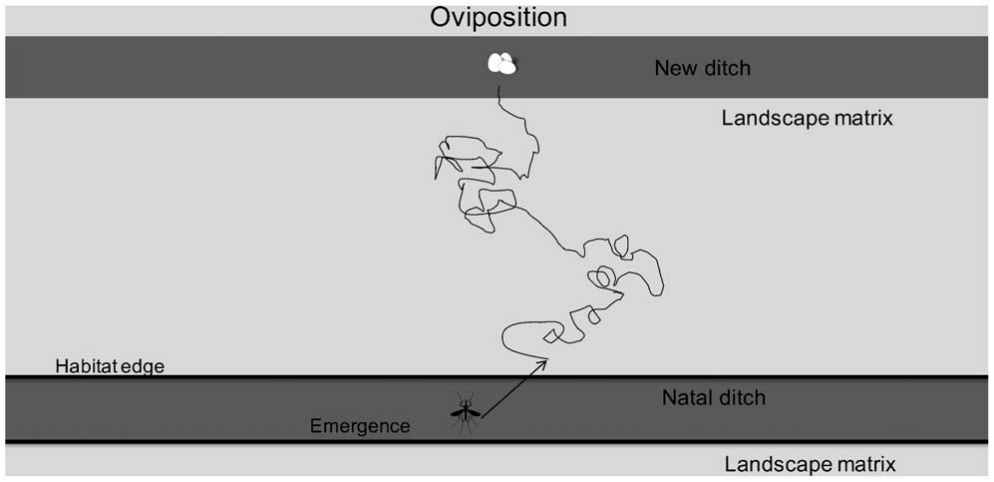
Simulated landscape in the movement model. The landscape consisted of the aquatic habitat, i.e. two ditches of 100 cells each, and the landscape matrix. The distance between the ditches, the landscape matrix and edge permeability varied among spatial scenarios. Distance between ditches amounted to 10, 20 and 30 m, edge permeability values were 0.001, 0.005, 0.01, 0.05 and 0.1, while matrix permeability values amounted to 0.2, 0.4, 0.6, 0.8 and 1.0.

**Figure 2:**
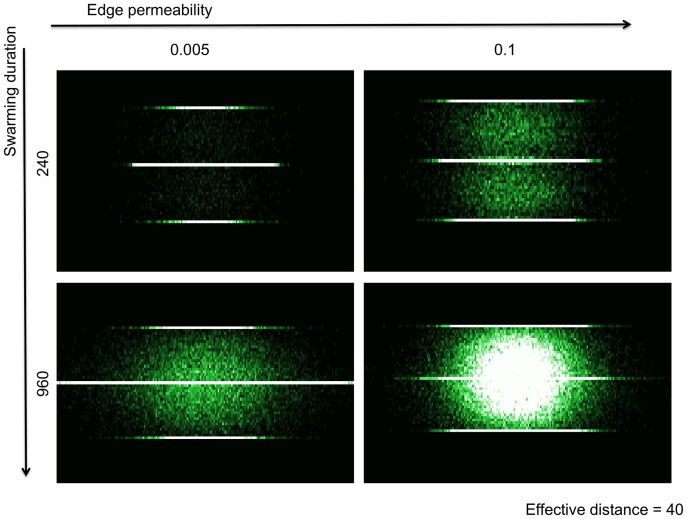
Density plots of individuals dispersing in the simulated landscape (effective distance was kept constant). Lighter colours depict higher densities (black cells harbour zero individuals). Low edge permeability limits dispersal outside of the natal ditch, whereas increasing the edge permeability and swarming duration allows individuals to laterally disperse.

The movement and spread of individuals in the simulated landscape (Figure 1) were governed by landscape-dependent movement parameters (Figure 2). We combined the landscape parameters, matrix permeability and distance between ditches, into one metric termed effective distance. When effective distance was kept constant, lower edge permeability resulted in linear dispersal along the natal ditch, whereas increasing the edge permeability resulted in individuals moving through the landscape matrix. The distances covered by moving individuals were enhanced with the increase of the swarming duration, i.e. obligatory movement before settling back into aquatic habitat (Figure 2).

**Figure 3:**
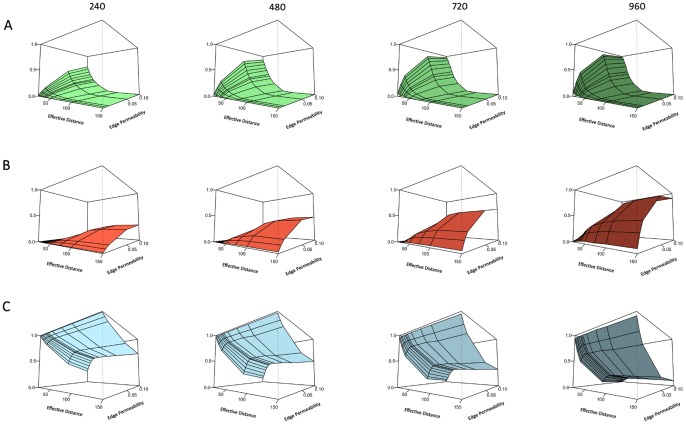
Outcome of the movement model in different landscape setups. A) probability of colonizing the new ditch, B) probability of mortality in the terrestrial habitat and C) staying in the natal ditch (z-axis; not labelled for better visibility of axis values). The probabilities are dependent on the landscape attributes. The surface plots of the output are separated into columns on the basis of the duration of the swarming movement, i.e. obligatory dispersal before individuals are allowed to settle in an aquatic habitat cell (240, 480, 720 and 960 minutes). Values on z-axes in all surface plots are from 0 to 1.0. Effective distances are plotted on the x-axis and span from 10 to 150 m, while the edge permeability values are plotted on the y-axis and span from 0.001 to 0.1.

Movement simulations yielded individual probabilities of successfully colonizing the other ditch, dying in the dispersal process and the probability to remain in or return to the natal ditch (Figure 3). Colonization probability increased mainly with smaller effective distance and higher edge permeability (Figure 3A), but also slightly with increasing swarming duration. Mortality especially increased with a combined increase in edge permeability and effective distance (Figure 3B), but also reached a higher level with longer swarming duration. With low edge permeability, mortality was low as most individuals were retained in their natal ditch. With small effective distances, dispersers always encountered a ditch (either the natal one or the other ditch). The probability of ending up in the natal ditch was consistently high for low values of edge permeability, but steadily decreased with increasing swarming duration at higher levels of edge permeability (Figure 3C). The increase of effective distance had less effect on the probability of staying, as it was mainly the edge permeability parameter that governed the process of leaving or staying in the natal ditch. However, in the case of permeable edges, the probability of staying in the natal ditch was very low for small values of effective distance (Figure 3C), due to the fact that many dispersers were trapped in the other ditch (competition between patches, see [34]).

### Midge Population Dynamics

**Figure 4:**
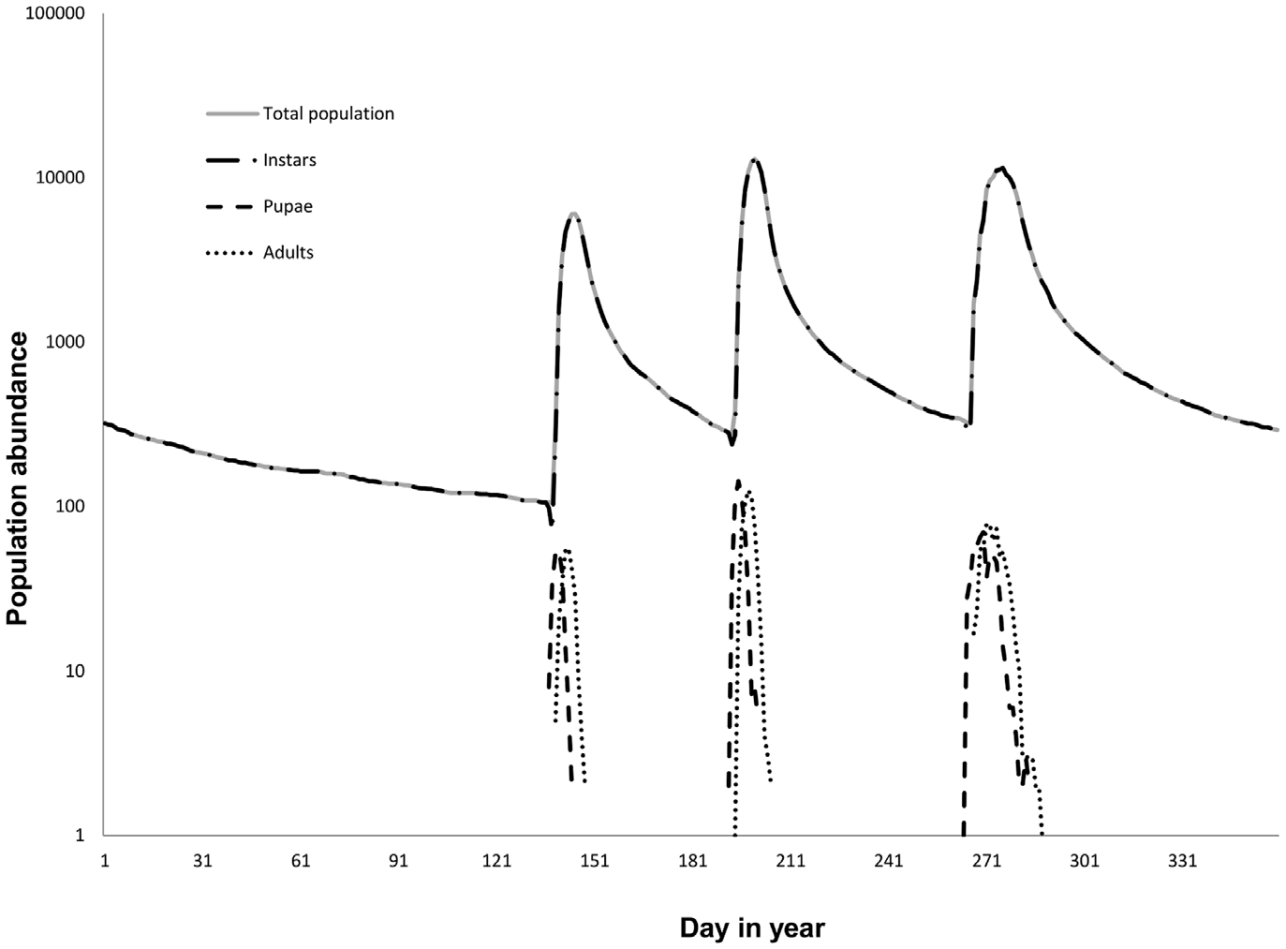
Simulated yearly dynamics of midge populations. Abundances are plotted on a log-scale. The population consists of larvae, pupae and adults, where larvae are most abundant. The larval abundance curve is indistinguishable from the curve depicting the dynamics of the total population.

Midge populations exhibited trivoltine yearly dynamics, i.e. had three generations (Figure 4). The population was dominated by larvae, abundances of which were controlled with density-dependent mortality. Consequently, the larval dynamics curves are indistinguishable from those of the total population (Figure 4).

**Figure 5:**
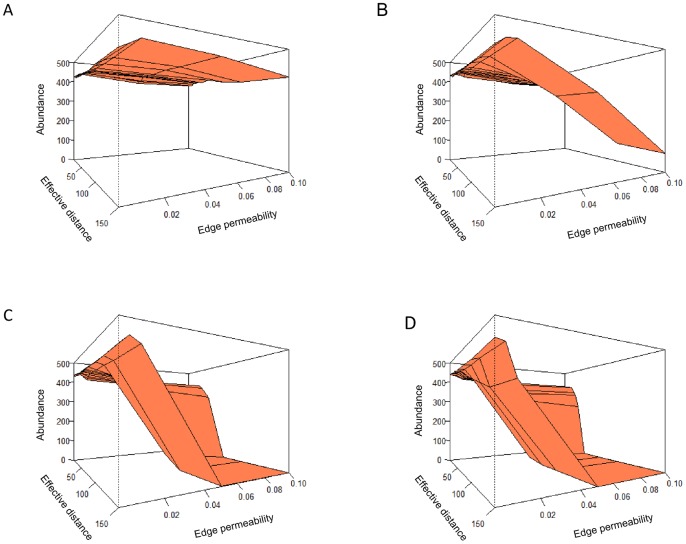
Medians of the total population abundance in different landscape and swarming setups. The daily population abundances were averaged over eight simulation years and 20 reference replicates, and were based on evaluated landscape parameters in the dispersal model. Each of the panels represents a different duration of the swarming movement, i.e. dispersal before individuals are allowed to settle in an aquatic habitat cell: A) 240, B) 480, C) 720, and D) 960 minutes swarming. Effective distances are plotted on the x-axis and the edge permeability values are plotted on the y-axis.

Because the dispersal parameters affected the abundances of modelled populations, we show here the median abundances for a range of landscape parameters evaluated in the movement model (Figure 5). The values in the surface plots were averaged over daily abundances of the total population (both ditches) of eight years of simulation and over the 20 reference replicates.

**Figure 6:**
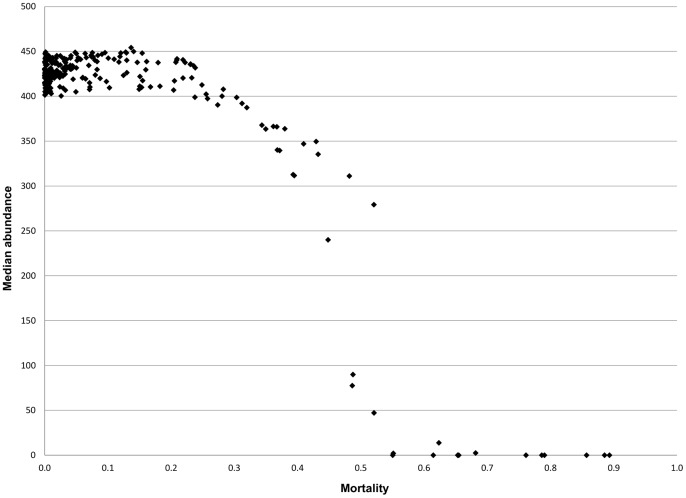
Realized median (reference) population abundances based on individual dispersal mortality. Most simulated populations became extinct when dispersal mortality of individuals amounted to 0.5.

Medians showed the same pattern as observed for survival probability (1– mortality, see Figure 3B). Combinations of landscape and movement attributes that lowered dispersal mortality risk consistently lead to higher (median) abundances. Higher dispersal mortality not only lowered abundances (Figure 6), but also lead to lower population viability, as above a certain dispersal mortality value (ca. 0.5), populations became extinct.

### Midge Population Recovery

**Figure 7:**
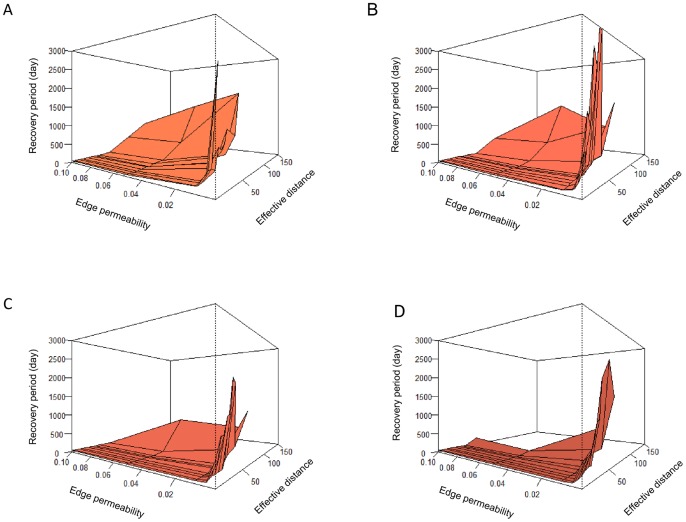
The median time to recovery of successfully recovered populations. Only accomplished recoveries were plotted. Each of the panels represents a different duration of the swarming movement, i.e. dispersal before individuals are allowed to settle in an aquatic habitat cell: A) 240, B) 480, C) 720, and D) 960 minutes swarming. Effective distances are plotted on the x-axis and the edge permeability values are plotted on the y-axis (note that the perspective of the surface plots is opposite than in previous figures).

Median time to recovery was shorter for high edge permeability and low effective distance (Figure 7, note that the perspective of the surface plot is opposite than in previous figures). Longer swarming duration further lowered the median recovery times. In a large part of the parameter space, with low edge permeability and/or high effective distance, no recovery was observed (median time longer than simulation duration).

## Discussion

Dispersal appears to be the limiting factor for colonization of stressed habitats, thus hampering the success of many restoration projects [5], [8]. We, therefore, evaluated different landscape attributes governing the dispersal and colonization potential of organisms.

Our results apply to any organism living in linear habitat elements, with limited dispersal ability, a period of obligatory movement, and running a considerable risk when venturing out of its reproduction habitat. We capture the main effects of landscape on movement, by accounting for a) a possible reluctance to leave the reproduction habitat (mediated by properties of e.g. vegetation at the interface between habitat and landscape matrix) [16], [19], [25], [28], b) effect of landscape matrix properties (e.g., vegetation, land-use) on movement, summarized in a possible slowing down of movement (smaller step length, lower velocity) [35], and c) the distance between linear habitat elements (patches).

Our results show that the combination of these three factors (where distance and matrix permeability can be collapsed into one metric, effective distance), determines the fate of an individual disperser. Individual fate can be summarized in three probabilities that are relevant at the (meta)population level, being the probability to end up in another habitat patch (ditch), to end up at another location in the same patch, or to perish in the landscape matrix. Though the differences in the outcome in terms of individual fate may not seem that large, with the population model we showed that for the (meta)population the balance between the probabilities of reproducing “here”, “there” or “not at all” (Figure 3– each column represents the balance among the three probabilities and sums to 1), had a huge impact on its abundance, viability and recovery potential (Figures 5, 6 and 7). The main insight from the movement model was, therefore, that all three probabilities may vary widely depending on movement and landscape attributes, and none of them can be ignored in (spatial) population modelling of this type of organism [36].

The population model output revealed that movement and landscape attributes can determine whether a population thrives in a landscape or not, and that this is mainly mediated by dispersal mortality risk (Figure 6). The balance between individual reproduction and mortality can shift in such a way as to lead to local populations with a high risk of becoming extinct, even in absence of any stress. Though this also implies that extinction risk for the whole metapopulation will increase, we should be aware that in reality metapopulation extinction risk depends on the number of patches (ditches) in the metapopulation [37] – while in the population model we address only the situation with two patches. The results of our study imply that there might be such extreme landscapes where chironomids or similar, dispersal limited, species might struggle to persist unless compensatory mechanisms, such as behavioural and/or genetic adaptations to extreme environments, are developed.

In our study, we have steered off the well-researched field of evolutionary trade-offs between dispersal and reproductive output [38], i.e. assuming different dispersal strategies in the population, mainly due to our firm focus on the interaction between specific movement ecology and landscape parameters and due to a very short timescale of our study. Furthermore, the population recovery potential in our study would have been heavily biased if we had introduced a trade-off between long and short distance dispersal. Therefore, in order to properly investigate the role of landscape on the population recovery potential, all individuals had been given the same initial chance and movement patterns as well as reproductive potential. Interestingly, our main results fall well in line with a study showing that, in populations with dispersal polymorphism, the invasion wave of polymorphic populations is fastest when both phenotypes, i.e. better dispersers or better reproducers, are present [39]. The combination of both dispersal potential, resulting in the (re)colonization probability and high reproductive output will ensure the fastest invasion wave, or, in our case, population recovery.

Due to lack of data on detailed movement patterns of our model organisms, we assumed a correlated random walk which was found to correctly describe insect movement [40] and to be the dominant strategy used by organisms in patchy environments [41]. We also simulated movement assuming the random walk (or Brownian motion), but also assuming Levy walks [42], both of which resulted in a similar, declining relationship of encountering another reproductive habitat with distance. However, the actual movement outcomes had an effect on the recovery and persistence of the metapopulation (results not shown). Assuming a random walk, individuals had lower colonization and higher mortality probability; whereas the opposite was true when assuming Levy walks. We, therefore, implemented a movement pattern in between those two extremes. Consequently, our model yields conservative estimates on dispersal potential of our model species, possibly underestimating distances that individuals may cover in real environments [24].

The way we defined the stress event, as an extermination of one of the local patch populations, implied that for recovery both re-colonization of the empty patch and growth of the re-established population are required. Re-colonization chances were high (Figure 3A) when effective distance between patches was low and edge permeability was high (or at least, not too small), i.e. when functional connectivity was high, which was consistent with many studies [7], [36], [43]. Population growth rate, resulting from the balance between reproduction and mortality, was largely determined by dispersal mortality, and thus highest when mortality was low (Figure 5 and 6). This was especially the case for low effective distance and/or low edge permeability, suggesting that very closed landscapes, e.g. those with thick riparian and other vegetation, successfully harbour high insect densities [29], [30], [44].

As a result of re-colonization chance and population growth rate, recovery times were, thus, expected to be shortest for the intersection of these areas in parameter space, i.e. where the combination of low effective distance and edge permeability above a threshold value allow for quick recoveries (Figure 7). This likely bears witness to the importance of re-colonization in the recovery process [8], [36] after such an extreme stress event.

### Landscape Management

Management of riparian vegetation is one of the priorities in many restoration projects [30], [31], [45] as it benefits the biodiversity and ecosystem functioning [46]. Such vegetation often represents a barrier for lateral dispersal thus limiting colonization potential of many species [5], [20], [25], as was also shown in our study. More recent insights from field and modelling studies on exchange of genetic material across landscapes imply that lateral dispersal occurs more often than previously expected [13], [21], [23], [24], however the data is still scarce and for many taxa the data is inconclusive [22].

We showed that, for the type of organism we modelled, landscape management aiming at high population abundances (Figures 5 and 6) should either promote a (very) low effective distance between habitat patches, OR a (very) low edge permeability, e.g. through riparian buffers. In other words, impeding insect movement into the landscape matrix by impermeable edges, unless ditches are quite near to each other and the matrix is highly permeable. On the other hand, if fast recovery of local populations is the main aim of landscape management, re-colonization is the key process and low effective distance AND high edge permeability are the factors promoting it. Thus, highly permeable edges, a permeable matrix and short distances are the aspects to focus on.

Focusing only on the management of vegetation (assuming distance between habitat patches is fixed) tells us that if the survival in the landscape matrix is relatively low, management should aim at promoting low edge permeability. Conversely, if the survival in the landscape matrix is sufficiently high, then the management should promote more permeable edges to allow for population exchange.

Our simulation study assumed landscape attributes that are constant throughout the year (and space), whereas many environments are seasonal. Consequently, the landscape matrix permeability may also vary within a year, e.g. in the thickness or height of the vegetation cover. In seasonally dynamic landscapes, aquatic insects have been found to disperse further in the landscape matrix early in the season, whereas thicker vegetation in other parts of the season retains many individuals closer to their natal habitats [25]. These findings are comparable with our results where the combination of landscape attributes, through impermeable edges and landscape matrix, benefited population abundance or, through increased functional connectivity and edge permeability, benefited dispersal. However, a more thorough analysis of this occurrence was beyond the scope of this study.

In conclusion, we showed that a combination of landscape attributes determines the fate of dispersing individuals. Once the individual probabilities to stay in the natal habitat, colonize a new habitat or perish in the landscape matrix are extrapolated to the level of a population, the balance between reproduction and dispersal mortality will have a big impact on population persistence. Furthermore, population persistence and population recovery benefit from landscapes where the effective distance between reproductive habitats is very low. However, the level of edge permeability will benefit either persistence (low permeability) or re-colonization and recovery (high permeability). Aims of landscape management may, therefore, need to be carefully considered and defined.

## Materials and Methods

We developed spatially explicit models of movement and population dynamics of the non-biting midge, *Chironomus riparius*. The population dynamics model makes use of dispersal rates derived from the movement model. Both models were programmed within the NetLogo platform [24]. Here we provide a short description of the movement and population model processes following the ODD protocol [47], while the full model description can be found in SI I. We also provide a sensitivity analysis of the population model (SI II).

### Movement Model

#### Purpose

The purpose of the movement model was to derive the rates and outcomes, i.e. probability of staying in the natal ditch, perishing in the landscape matrix and probability of reaching the new ditch, after movement in landscapes of differing permeability.

#### State variables and scales

The entities in the movement model were adult chironomids and landscape cells.

Chironomid state variables included the turning angle [degrees], the step size [cm] and their location [continuous X and Y coordinates]. Chironomids are considered to be relatively weak flyers, often carried by the wind [48]. We simulated their dispersal patterns by assuming the correlated random walk (CRW) [41], [49]. Correlated random walks combine a non-uniform distribution of turning angles with an exponentially decaying distribution of step lengths. Here we used the von Mises angular distribution [50], i.e. a normal distribution on a circle, in which we vary the degree of angular correlation by altering the shape parameter, *κ*. We set the mean value to 0 and the value of *κ* to 6 which translates into fairly correlated movement. Every time step, each adult individual was assigned a turning angle and a step from respective distributions (see Table 1 for details). Given the tiny size of our model organisms, we assumed one minute as a simulated time step, and a total dispersal period of 16 hours (960 minutes). Adult individuals were, thus, assumed to disperse for less than one day; this is a simplification from the findings of Downe [51] who showed that males are reproductively active only for two days.

**Table 1:** Life-history parameters of *Chironomus riparius* used in the individual-based model and the correlated random walk (CRW) parameters used in the movement model.

Parameter	Value	Unit	Interpretation	Reference
***μ_0_***	0.0007	/	Daily, background mortality probability	Calibration estimate; expert opinion
***μ_1_***	0.003	/ind	Density-dependent mortality scaling factor	Adapted from [55]
***fecundity***	50–150	eggs	Individual fecundity	Adapted from [54], [60]; corrected for modelling only females, assuming 1∶1 sex ratio
***egg stage***	5	day	Duration of the egg stage	Expert opinion; adapted [33], [61]
***pupal stage***	2	day	Duration of the pupal stage	Expert opinion; adapted [33], [61]
***l_0_***	mean 0.002, SD 0.0001	mm	Normally distributed individual length at hatching	Adapted from [54]
***γ***	0.095	/day	Individual growth rate	Calibration estimate; based on ca 15 days that it takes to reach maximum size of larvae before pupation
***l_max_***	13.72	mm	Normally distributed maximum length	[54]
***turning angle***	mean 0, *κ 6*	°	Von Mises distribution	Assumption
***step duration***	12	steps	Mean of the exponential distribution	Assumption
***step length***	2	cm	fixed step length	Assumption

Landscape cell variables included the edge and landscape matrix permeability. The simulated landscape was comprised of aquatic habitat, i.e. ditches consisting of a string of 100 cell, separated by the terrestrial (non-)habitat, in the following referred to as the landscape matrix (Figure 1). Each cell represented one square meter, thus making the ditch 1 m wide, comparable to a Dutch agricultural landscape. A large number (10000) of chironomid movement paths was generated, all starting from the centre of one ditch (natal ditch in Figure 1). The landscape impacted movement through edge and matrix permeability. Edge permeability refers to the probability of crossing the border between the natal ditch and landscape matrix for a movement path that ‘hits’ this edge from the inside of the ditch. Note that the probability of crossing this edge in the opposite direction (into the ditch) was set to 1, as we assume no reluctance in entering the reproductive habitat (as opposed to assumed reluctance in leaving it). Matrix permeability refers to the extent to which the landscape facilitated movement (the reciprocal of ‘resistance’), and was represented by a scaling factor on realized step size.

#### Process overview and scheduling

Chironomid movement continued for the full dispersal period, unless water (a ditch) was encountered. In that case, movement halted, but only outside the period of ‘obligatory’ movement, the swarming period. Dispersers that did not encounter any water at all during the dispersal period were assumed to perish in the landscape matrix (dispersal mortality). Note that dispersers that encountered another than the natal ditch were always allowed to settle, even during the swarming period. Having a swarming period is characteristic for many aquatic insect species [52], wherein the males form swarms and mate with passing females. In our study, swarming is equated with an obligatory part of the movement, before individuals are allowed to settle back into the aquatic habitat. The critical parameters in the simulations were the duration of the swarming period, the distance between ditches and the permeability of the edge and the matrix. The duration of the swarming period was set to 240, 480, 720 and 960 time steps, i.e. minutes. Distance between ditches amounted to 10, 20 and 30 m. Edge permeability values were 0.001, 0.005, 0.01, 0.05 and 0.1, while matrix permeability values amounted to 0.2, 0.4, 0.6, 0.8 and 1.0. Our definition of matrix permeability allowed us to combine matrix permeability and distance between ditches in one metric, effective distance ( = distance/matrix permeability).

Each movement simulation produced three probabilities for use in the population model when run for the same spatial settings: 1) probability of staying in (or returning to) the natal ditch, 2) probability of encountering the other (“new”) ditch (functional connectivity), and 3) probability of dying after the dispersal period ends (not encountering the aquatic habitat). In addition, for dispersers ending up in one of the ditches, from their x-coordinates one-dimensional dispersal kernels were estimated, defining the probability of covering a certain distance within a ditch. Note that given the setup for the movement model (movement starting from one ditch in a two-ditch landscape with periodic boundary conditions, Figure 1) the results refer to a wrapped-around landscape, where each ditch will have another ditch on both sides, and in longitudinal direction each ditch cell will have another neighbouring ditch cell on both sides. The probabilities of movement to the other ditch and mortality in the matrix obtained from the movement model were summed for movement in left- and right direction from the natal ditch, and used in the population model with identical spatial structure (Figure 1).

### Population Model

#### Purpose

The purpose of the model was to simulate the population dynamics of the non-biting midge, *Chironomus riparius*, and to evaluate the re-colonization potential and population recovery after extreme stress in relation to the landscape specific parameters.

#### State variables and scales

The entities of the model were the female individuals and the landscape. Chironomid females were characterized by the following state variables: age [days], developmental stage [larva, pupa or adult], body size of larvae [length in mm], reproductive status and fertility of female adults [number of offspring], dispersal status and their location [X and Y coordinates].

The simulated landscape consisted of square cells on a 2×50 grid, of which 100 cells represented the aquatic habitat. These cells formed two ditches, each consisting of a string of 50 cells. The state variables of ditch cells were the stress induced mortality probability and the mortality as a consequence of density of individuals within one cell.

Only one ditch was subjected to stress. Treated populations were assumed to undergo extreme stress (100% mortality in the treated ditch) on day 150 (1^st^ June); such extreme stress could represent a restoration or colonization effort of a newly built ditch.

The basic time step in the model was one day. The number of days in a month was always assumed to be 30; there were thus 360 days in a year. The simulations ran for nine years or until there were no surviving individuals left. The first year of simulation was discarded to avoid transitional effects in the output, leaving, thus, eight simulation years for analysis. Processes in the model were executed in a prescribed order, but randomly within the population. All parameters and their distributions are provided in Table 1.

#### Process overview and scheduling

The model included different processes for different life stages of a chironomid population. Here we described the basic life-history as implemented in the model.

Only female individuals were modelled. The life-cycle started with the larval stage, which contained an inactive phase, mimicking the egg life stage (five days). Active larvae grew according to a temperature-dependent von Bertalanffy growth function [53]. Once the larvae reached their maximum size, they pupated and stayed in this stage for two days after which individuals emerged as adults. Based on the dispersal simulation results (thus depending on ditch distance and landscape permeability values), dispersing adults had a probability of staying in the natal ditch, of moving to the other ditch and of dying in the landscape matrix. If an adult female was successful in dispersing (found a suitable aquatic habitat), she deposited a certain number of eggs/inactive larvae; the number of eggs/inactive larvae was drawn from a uniform distribution, the number of which was corrected for modelling only females (assuming 1∶1 sex ratio). From here, the life-cycle started from the beginning.

#### Density-dependence

Including density-dependent mortality is an indirect way of modelling resource competition, as we do not explicitly account for resource dynamics in the population model. Chironomid populations are weakly regulated by their densities; populations can attain very high densities before density-dependent consequences are visible. Pery et al. [54] show that individual growth is hampered by increasing densities in their experimental system, with first effects visible at 10 individuals per beaker (14 cm^2^).

In the population model, a linear increase in mortality was assumed (based on [55], see SI I for more detail). This was based on the density of individuals within one cell where each individual had a certain effect on its conspecifics, governed by the mortality scaling factor (Table 1).

#### Temperature-dependent growth

Water temperatures, an exogenous process, were used as an input to our population model, and were based on year round data collection from ditches in the Netherlands [56]. Temperatures changed on a daily basis, but were kept equal for all cells in the modelled landscape, and no interannual variation was assumed (see SI I for more details). The growth function of larvae was set up in such a way that the increment in individual size (mm) exponentially increased with rising water temperatures (adapted from [57]), with a maximum increment at a water temperature of 24°C [58], [59].

Since the temperatures governed larval growth which regulated the generation time, the number of generations in one year was an emergent property of the model (the analysis of the temperature dependency is described in SI II).

### Analysis of Population Recovery Times

The model output from populations exposed to stress was compared with that from control populations. For the analysis of recovery times after each of simulated exposures, we used 20 replicate simulations of treatment and control. Daily abundances of 20 treated populations were compared to 20 replicates of control populations, yielding potentially 400 recovery times. A treated population was considered to be recovered once its abundance reached or was higher than 95% of abundance of the control population; if this condition was met for five days within a ten day period, we deemed the population recovered. The day of recovery was then noted to be in the middle of this 10 day period. Finally, each parameter combination gave a distribution of recovery times, i.e. maximum of 400 values, of which we used the median value for further analysis and plotting.

## Supporting Information

Figure S1 **Effects of increasing water temperatures (deltaT = 2) on the number of generations in the modelled population. Only the third year of the simulation is plotted.**
(TIF)

Figure S2 **Total population abundsance (log10) as a result of increasing water temperatures. Boxplots represent abundances of three simulation years.**
(TIF)

Figure S3 **Effects of the density-dependent factor on total population abundances (log10 scale).**
(TIF)

Figure S4 **Effects of the density-dependent factor on population recovery time.**
(TIF)

Supporting Information SI **Description of the Chironomus riparius population model.**
(DOCX)

Supporting Information SII **Sensitivity analysis of the Chironomus riparius population model.**
(DOCX)
