# Supplementary figures and images for: Persistence of Aquatic Insects across Managed Landscapes: Effects of Landscape Permeability on Re-Colonization and Population Recovery
Source: PLoS One. 2013 Jan 24;8(1):e54584. doi: 10.1371/journal.pone.0054584 (PMC3554752; doi:10.1371/journal.pone.0054584)

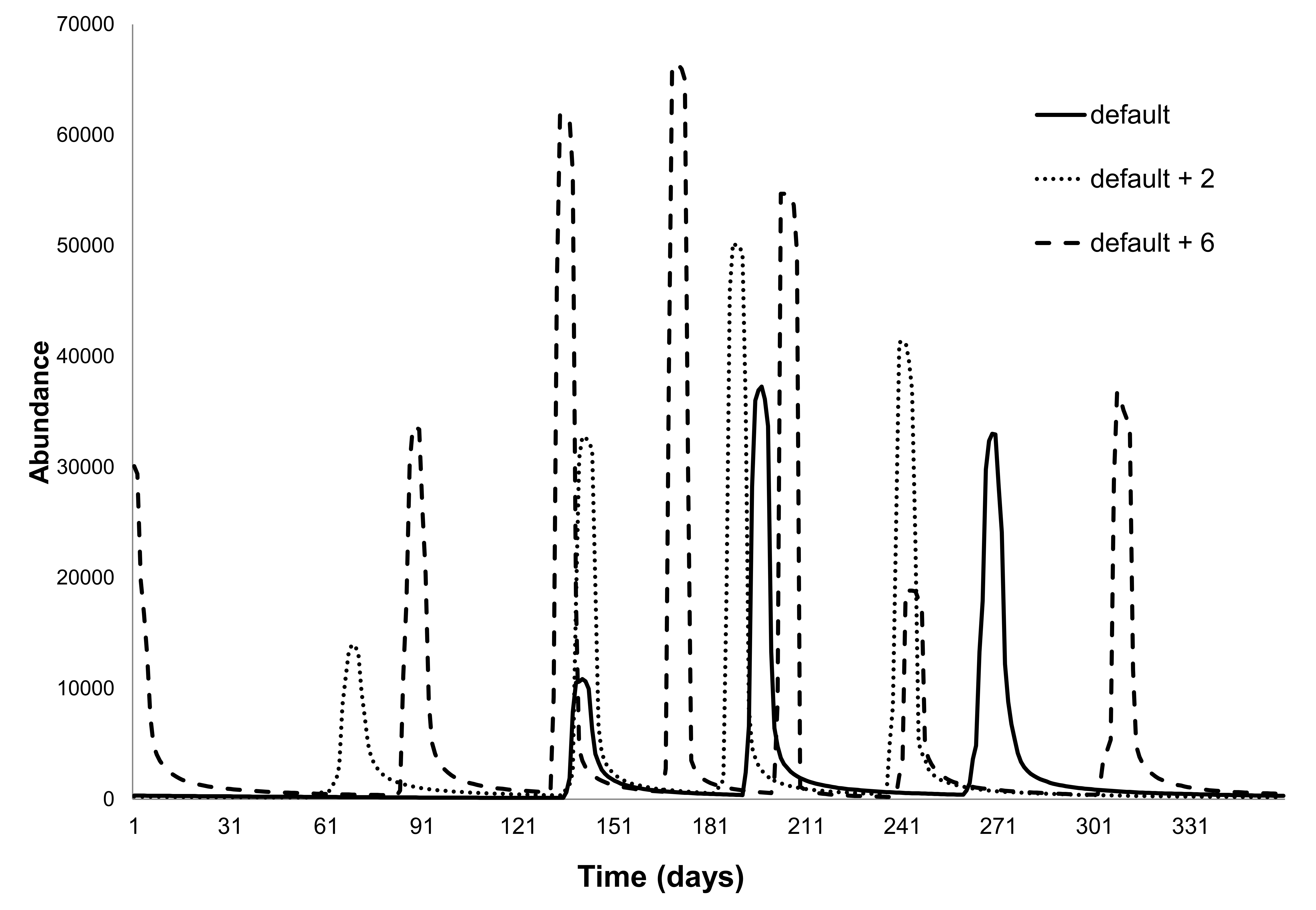

Supplement: Figure S1 — Effects of increasing water temperatures (deltaT = 2) on the number of generations in the modelled population. Only the third year of the simulation is plotted. (TIF) [file pone.0054584.s001.tif]

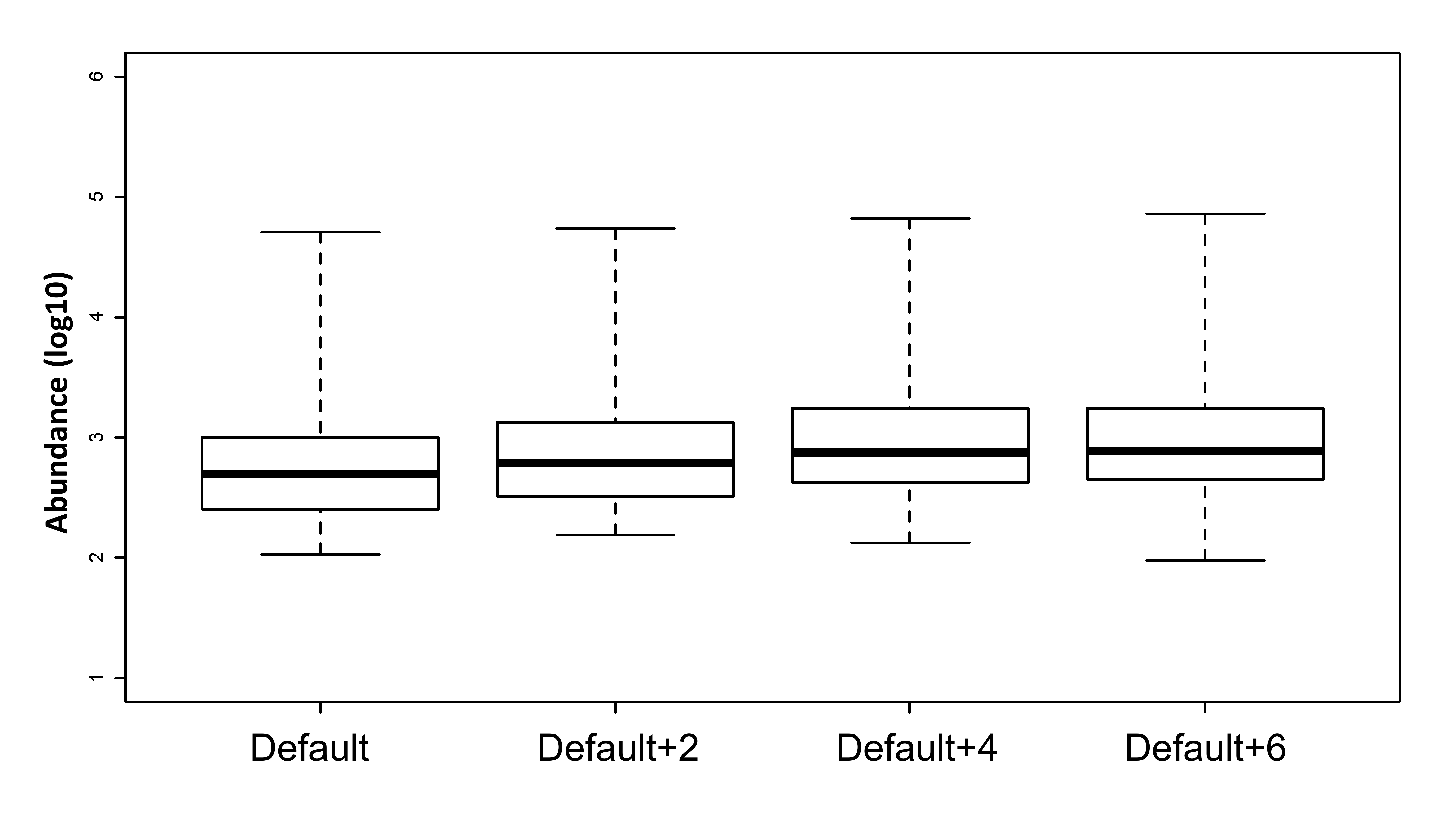

Supplement: Figure S2 — Total population abundsance (log10) as a result of increasing water temperatures. Boxplots represent abundances of three simulation years. (TIF) [file pone.0054584.s002.tif]

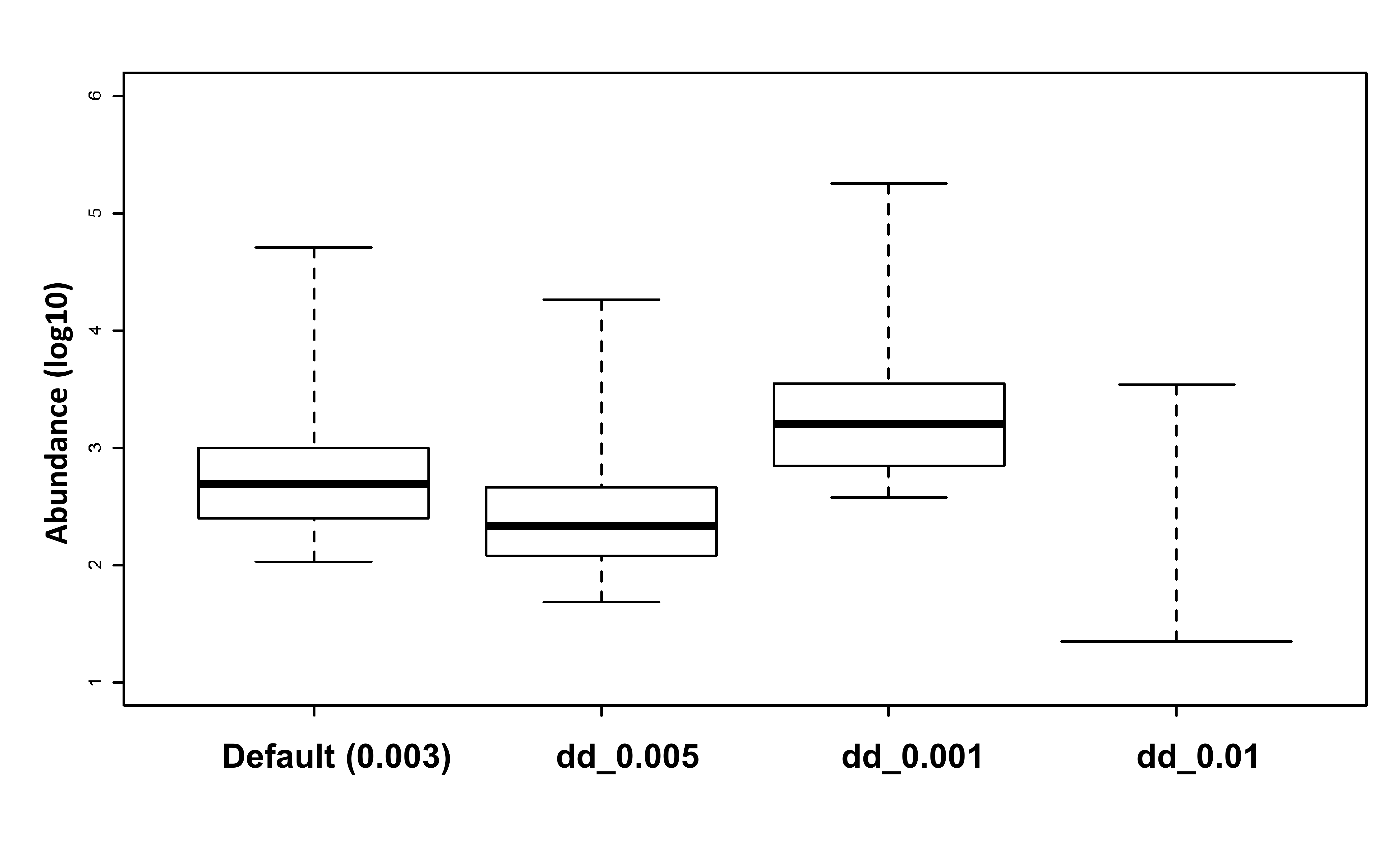

Supplement: Figure S3 — Effects of the density-dependent factor on total population abundances (log10 scale). (TIF) [file pone.0054584.s003.tif]

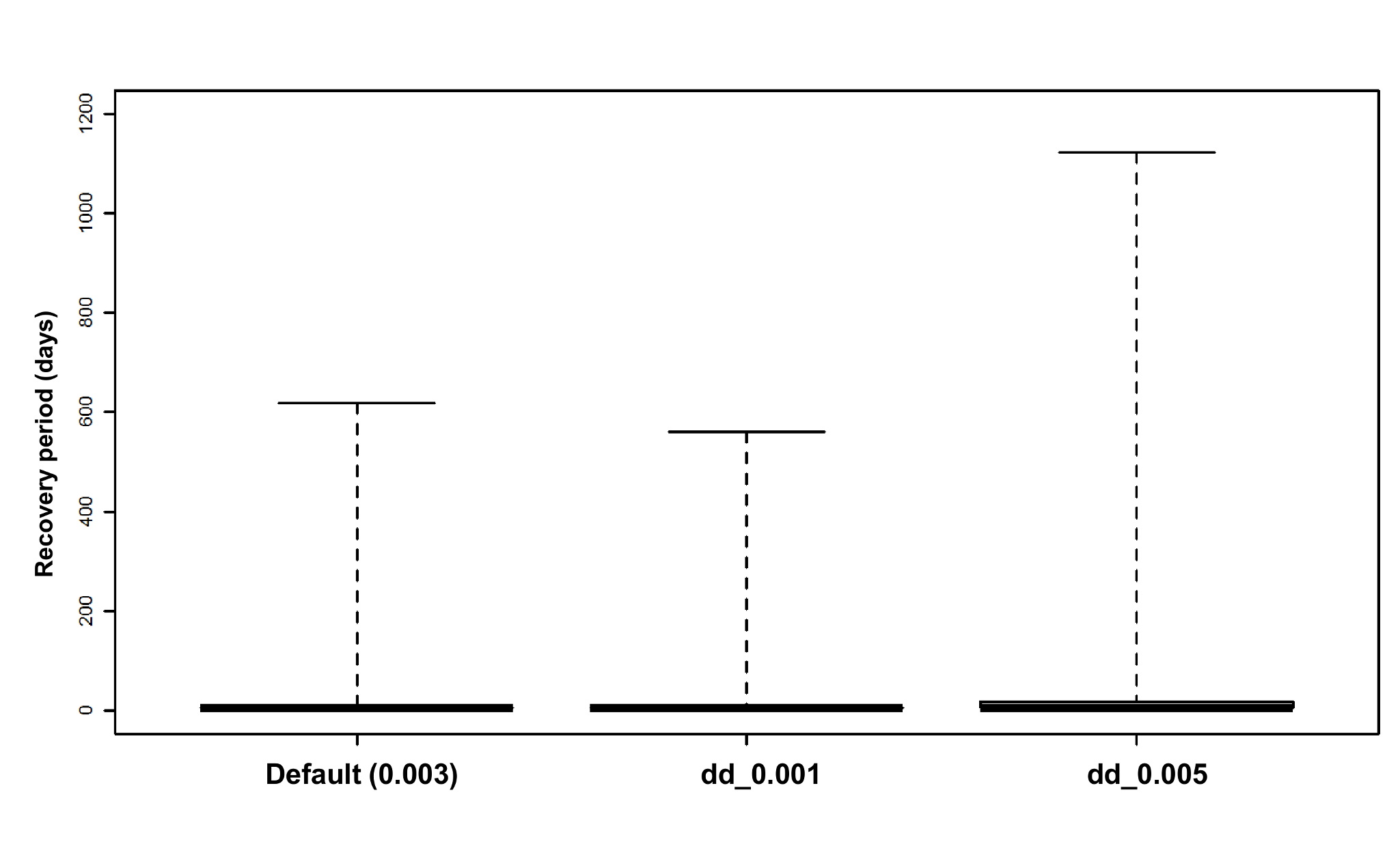

Supplement: Figure S4 — Effects of the density-dependent factor on population recovery time. (TIF) [file pone.0054584.s004.tif]
