# Supplementary material for: Persistence of Aquatic Insects across Managed Landscapes: Effects of Landscape Permeability on Re-Colonization and Population Recovery
Source: PLoS One. 2013 Jan 24;8(1):e54584. doi: 10.1371/journal.pone.0054584 (PMC3554752; doi:10.1371/journal.pone.0054584)
Supplement: Supporting Information SI — Description of the Chironomus riparius population model. (DOCX) [file pone.0054584.s005.docx]

**Supporting Information I**

**Description of the *Chironomus riparius* population model**

The model description follows the ODD protocol for describing individual- and agent-based models [1]. This document describes version 1.401 of the population model (ChD_v1.401).

**Overview**

Purpose

The purpose of this model is to simulate the population dynamics of the non-biting midge, *Chironomus riparius*, for evaluating population persistence and recovery after stress in managed landscapes. Both persistence and recovery are assessed in relation to the permeability of the landscape matrix, edge permeability and distance between the aquatic habitats. Dispersal in the landscape is simulated with a separate movement model.

State variables and scales

The entities of the model are habitat cells and female individuals.

The chironomid females are characterized by the following state variables: developmental stage [egg, larval, pupal and adult stages], body size of larvae [mm], reproductive status and fertility of females, dispersal status and their location [continuous X and Y coordinates].

The environment is a 2x50 cell grid, where 100 cells are aquatic habitat. These cells form two ditches, each consisting of a string of 50 cells (Figure 1). The state variables of ditch patches are the mortality probability induced by pesticides and by the density of individuals in the patch. Only one ditch is treated with pesticides, while the second one serves as the source of individuals needed for recolonization (Figure 1).

The basic time step in the model is one day. There are 360 days in a year. The simulation runs for 9 years or until there are no surviving individuals left. The first year of the simulation is discarded to avoid transitional effects in the output, leaving, thus, eight simulation years for analysis. Processes in the model were executed in a prescribed order, but randomly within the population. All parameters are listed in Table 1.

The model is programmed within the NetLogo platform [2].

Process overview and scheduling

The model includes different processes for different life stages of a chironomid population.

Here we first describe the basic life-history of the species as implemented in the model, followed by the pseudo-code.

Only female individuals were modelled. The life-cycle started with the larval stage, which contained an inactive phase, mimicking the egg life stage (five days). Active larvae grew according to a temperature-dependent von Bertalanffy growth curve [3]. Once the larvae reached their maximum size, they pupated and stayed in this stage for two days after which individuals emerged as adults. Based on the movement model output, dispersing adults ended up staying in the natal ditch, moving to the other ditch or dying in the landscape matrix. If an adult female was successful in dispersing (found a suitable aquatic habitat), she deposited a certain number of eggs/inactive larvae; the number of eggs/inactive larvae was drawn from a uniform distribution where the amount was corrected for modelling only females, assuming a 1:1 sex ratio. From here, the life-cycle started from the beginning.

The following pseudo-code gives an overview of the processes and their schedule, which is run each day and, depending on the life stage distribution in the population, is differentially activated.

Update age of individuals by one time step

Update water temperatures

*If* **mortality** of juveniles

Delete from the population

*Else* **hatch from egg** – for inactive larval stage

**Grow** – for active larval stages

**Pupate** - ditto

**Emerge** – for pupal stages

**Disperse** – adult females (relevant parameters obtained from the

movement model)

**Oviposit** – once they found a suitable habitat patch, females will oviposit

**Stress induced mortality**

Changes in state variables caused by the model processes are updated immediately.

**Design Concepts**

*Emergence.* Population dynamics and spatial arrangement of individuals emerges from the model rules. Number of generations is not imposed, it is based on individuals’ growth and reaching the threshold size for reproduction. Individual growth is dependent on water temperatures with a consequence that higher temperatures lead to shorter generation time, yielding more generations per year (see SA of the model, SI II).

*Interaction.* Individuals interact indirectly via local density-dependent effects on mortality. Probability of dying increases with increasing density of individuals in a local cell.

*Stochasticity.* Parameter values for the initial larval size and fecundity are drawn from probability distributions obtained from literature data to represent natural variability observed in chironomid populations. Turning angle and step size distributions in the movement model are taken as best conservative assumptions based on the reviewed literature. The outputs of the movement model and individual daily mortality are stochastic processes. All parameters are listed in Table 1.

*Observation.* For model testing, the behaviour of single individuals and of the whole population was observed. The dynamics of the total and of the population in only the treated ditch, averaged growth of individuals, population size distribution, population death rate, temperature dependent growth rate, abundance of adult females were all observed.

**Details**

*Initialization.* The model is initialized with a given number (10 per patch) of individual larvae. All individuals represent an active larval form (inactive being the egg stage). Individuals are distributed randomly in a ditch. Each initialization starts with the same number of individuals, but with different spatial coordinates and state variable values, e.g. body size (mean 3.5, SD 0.5 mm, based on sizes of overwintering larvae [4].

The pseudo-code gives an overview of the initialization process

*to initialize*

initialize global parameters

initialize landscape [ set carrying capacity

set pesticide induced mortality probability …

initialize chironomids [ set size

set age

set location …]]

*end*

*Input.* External (water) temperatures are used as a forcing factor for individual growth. Temperatures are based on year round data collection from ditches in the Netherlands [5]. The temperatures in the model change, therefore, in each time step within one simulated year, but no interannual differences are assumed.

*Submodels*. Submodels include: mortality of aquatic and terrestrial stages, individual growth and dispersal with oviposition.

Mortality of aquatic stages

There are three different mortalities affecting individuals in aquatic part of the life-cycle, background or natural mortality, density-dependent and stress-induced mortality. We assume daily mortality rates only for the larval stage (eggs and pupae have 100% survival).

Including density-dependent mortality is an indirect way of modelling resource competition, because we do not model resource dynamics. Even though exact mechanisms of density dependence are not clear, it is known that populations of chironomids are regulated by their densities, although these densities can be very high before any regulation occurs, e.g. Pery et al. (2002) [6] show that animals in beakers with higher numbers of individuals grow slower due to food limitation (beakers have a surface area of 14 cm^2^ and the effects on growth are visible already with 10 individuals in one system). With ad libitum feeding conditions, density dependent effects seem to be much less pronounced. Hooper et al. (2003) [7] also show that at densities of 16 individuals/cm^2^, only 1% of individuals eventually emerges.

The realized densities in the model are kept lower than observed in the natural environment, in order to decrease the simulation time. Within this model, we assume that individual mortality is based on local densities [8]

$\mu_{dd}=\mu_{1}\cdot N$ eq. 1

where $\mu_{1}$ is a parameter governing the steepness of the density dependence (m^2^/ind*d) and *N* is the local density (ind/m^2^). Density-dependent mortality is cell based, so all individuals in one cell have the same probability of dying due to overcrowding.

Eq. 1 is the simplest assumption on effects of density, where each individual has the same effect on each of its conspecifics within a cell. We test the model output with regards to different values of $\mu_{1}$(see SI II for details).

An additional mortality implemented in the model is the winter mortality of larvae. It has been observed that only fourth instar chironomid larvae survive the winter period [9]. We, thus, assume that all individuals smaller than 3.5 mm (M. Marinkovic *personal communication*) do not survive the winter period and are removed from the population.

Mortality of terrestrial stages

In the model, adult females may suffer from dispersal mortality. If they survive the dispersal routine and oviposit in one of the ditches, they die immediately after oviposition.

Individual growth

This species’ growth and development is highly dependent on available food and temperature of the surrounding environment. It can be uni- to multivoltine, depending on local environmental conditions [5,10]. In this model, the population has three generations in a year, found also in Eggermont and Heiri and Learner and Potter [10,11].

With an ad libitum feeding regime, isomorphic larvae grow according to von Bertalanffy growth curve [3]

eq. 2

where *l_max_* is the maximum larval size after which individuals pupate, and *γ* is the daily growth rate. With the given individual growth rate (Table 1), larvae finish their growth within approximately 12 days (temperature independent).

Differentiating the growth function, we obtain the daily increment in growth

$\frac{dl}{da}=\gamma\cdot l\cdot(\frac{l_{\max}}{l}-1)$ eq. 3

Growth is dependent on water temperatures, which change on a daily (time step) basis, and is implemented as

$TD=\frac{1}{10\cdot e^{-0.1\cdot externaltemperature}}$ eq. 4

This is then multiplied with the daily growth increment

$increment=\gamma\cdot l\cdot(\frac{l_{\max}}{l}-1)\cdot\frac{1}{10\cdot e^{-0.1\cdot externalTemperature}}$ eq. 5

and added to the current size of the individual. The function is set up in such a way that the size increment exponentially increases with rising water temperatures, e.g. with the water temperature of 24° C, the increment added is maximal and eq. 4 reaches a plateau [10]. This was also tuned to laboratory data on chironomid growth, where water temperatures are kept at a constant 21° or 25°; when the individuals are kept at 21°, one generation takes approximately 35 [12], whereas in the model it takes ca. 42 days.

Modelled individuals do not grow at all if the temperatures are lower than 8 degrees (adapted from [9]). The maximum value in the water temperature values used in this model is 18.8° C.

Reproduction

Each adult female that survives the dispersal process oviposits a number of eggs; the value is drawn from a uniform distribution (Table 1), and corrected for modelling only females, assuming a 1:1 sex ratio.

Movement and dispersal

Generally, chironomids are relatively weak dispersers that cover bigger distances with the help of wind [13]. Delettre and Morvan [14] showed that the number of trapped chironomids drop significantly (exponential decrease) with the distance from the nearest water body. Their results also suggest that adults accumulate in nearby hedges for resting, their dispersal is therefore hampered. Furthermore, it seems that dispersal distances can be linked to seasonal foliage cover of trees, where earlier in the year, more organisms can be found further, due to low tree cover and, thus, less barriers. The limited lateral dispersal is corroborated also by the findings of, amongst many others, Petersen [15] who find the majority of adult mayflies and caddisflies move no further than 7 – 11 m from the stream they emerged from. Smith et al. [16] review the dispersal of aquatic insects in the context of restoration efforts, and find the preference of adults for vegetated areas. Chironomid dispersal usually includes 3 types of movement: initial dispersal after emergence to the resting site, swarming behaviour and ovipositing flight of females [17]. Briggs and Latto [18] show that a species of gall-forming midge disperses from the point of release on average only about 1.7 m.

In a separate model, we simulate movement of a large number of individual chironomids and analyse three main factors relevant for their impact on individual survival and colonization probability: edge permeability, landscape permeability, and distance between two ditches. We assume individual chironomids move following the correlated random walk.

Here we provide a description of assumptions and processes in the movement model.

**Individual movement patterns**

We simulate individual movement of flying insects, assuming a correlated random walk (CRW). Correlated random walks (CRW) [19,20] combine a non-uniform distribution of turning angles with an exponentially decaying distribution of step lengths. Here we used the von Mises angular distribution [21], i.e. a normal distribution on a circle, in which we vary the degree of angular correlation by altering the shape parameter, *κ*, which is the measure of concentration for this distribution and is analogous to $\frac{1}{\sigma^{2}}$of a normal distribution. Smaller values of *κ* diffuse the distribution, while it becomes a uniform distribution at value 0, resulting in Brownian motion (also random walk). Larger values of *κ* result in the distribution centring more on the mean, which means that the movement will be more directed. Here we set the mean value to 0 and the value of *κ* to 6 (fairly correlated movement).

We assign individuals with an initial step size of 2 cm. Every time step, each adult individual was assigned a turning angle and a step from respective distributions (see Table 1 for details). Given the tiny size of our model organisms, we assumed one minute as a simulated time step, and a total dispersal period of 16 hours (960 minutes). Adult individuals were thus assumed to disperse for less than one day; this is a simplification from the findings of Downe [23] who showed that males are reproductively active only for two days.

**Movement model**

The simulated system consists of two ditches, separated by the landscape matrix (Figure 1), which presents a certain risk for flying adult females, which we do not explicitly model, but assume the individuals die if they end up in the matrix by the end of simulation time.

The landscape impacted movement through edge and landscape matrix permeability (Figure 1). Edge permeability refers to the probability of crossing the border between the natal ditch and landscape matrix for a movement path that ‘hits’ this edge from the inside of the ditch. The probability of crossing this edge in the opposite direction (into the ditch) was set to 1, as we assume no reluctance in entering the aquatic, i.e. reproductive, habitat (as opposed to assumed reluctance in leaving it).

The landscape matrix permeability refers to the extent to which the landscape facilitated movement (the reciprocal of ‘resistance’), and was represented by a scaling factor on realized step size. This factor ranges between 0.1 and 1, where the latter denotes a fully permeable landscape.

The lower the parameter, the longer it will take an individual to reach the destination ditch. Permeability of vegetated cells is a parameter tuning the distances covered by dispersing individuals. Individuals are assigned an initial step size, set to 2 cm. This step size is then modified based on the habitat permeability parameter of each cell. The final value will be the actual distance covered in that time step.

Instead of a typically used step size distribution for the random walk model, we use distribution of times spent in movement, i.e. the number of steps made within the simulated time step of 1 minute.

**Example**: an individual is assigned a direction of movement (from the von Mises distribution) and a value of 12, which is the mean of the movement time distribution, representing 12 steps that will be covered within one simulated time step, thus not changing direction. Based on the permeability of habitat cells the individual crosses, the fixed step size, i.e. 2 cm, can be smaller or bigger (but not bigger than the initial value). In very impermeable landscapes (permeability parameter of 0.1), this will result in an individual crossing 2.4 cm (0.1*2*12), whereas in very permeable habitat (permeability of 1), an individual will cross 24 cm in one minute.

We assume that individuals stop immediately as they reach the new ditch and that females can sense the water cells in a certain radius and move directly towards it at the end of the simulation.

As a result of dispersal simulations, each scenario yields 7 parameters:

1. probability of staying in the native ditch
2. probability of moving to the new ditch
3. probability of dying during the dispersal process
4. mean location in the native ditch
5. standard deviation of the location in the native ditch
6. mean location in the new ditch
7. standard deviation of the location in the new ditch

Finally, for analysis of results and interpretation of model outcomes in the manuscript, we combined matrix permeability and distance between ditches into one metric, effective distance (= distance / matrix permeability).

References

1. Grimm V, Berger U, Bastiansen F, Eliassen S, Ginot V, et al. (2006) A standard protocol for describing individual-based and agent-based models. Ecological Modelling 198: 115-126.

2. Wilensky u (1999) NetLogo. Centre for Connected Learning and Computer-Based Modelling, Northwestern University, Evanston, Il.

3. von Bertalanffy L (1957) Quantitative Laws in Metabolism and Growth. The Quarterly Review of Biology 32: 217-231.

4. Huryn AD (1990) Growth and voltinism of lotic midge larvae: patterns across an Appalachian mountain basin. Limnology & Oceanography 35: 339-351.

5. Veraart AJ, de Klein JJM, Scheffer M (2011) Warming Can Boost Denitrification Disproportionately Due to Altered Oxygen Dynamics. PLoS ONE 6: e18508.

6. Pery ARR, Mons R, Flammarion P, Lagadic L, Garric J (2002) A modelling approach to link food availability, growth, emergence, and reproduction for the midge *Chironomus riparius*. Environmental Toxicology and Chemistry: 2507-2513.

7. Hooper HL, Sibly RM, Hutchinson TH, Maund SJ (2003) The influence of larval density, food availability and habitat longevity on the life history and population growth rate of the midge Chironomus riparius. Oikos 102: 515-524.

8. Galic N, Baveco H, Hengeveld GM, Thorbek P, Bruns E, et al. (2012) Simulating population recovery of an aquatic isopod: Effects of timing of stress and landscape structure. Environmental Pollution 163: 91-99.

9. Rasmussen JB (1984) The life-history, distribution, and production of Chironomus riparius and Glyptotendipes paripes in a prairie pond. Hydrobiologia 119: 65-72.

10. Eggermont H, Heiri O (2011) The chironomid-temperature relationship: expression in nature and palaeoenvironmental implications. Biological Reviews 87 (2): 430–456.

11. Learner MA, Potter DWB (1974) The seasonal periodicity of emergence of insects from two ponds in Hertfordshire, England, with special reference to the chironomidae (Diptera: Nematocera). Hydrobiologia 44: 495-510.

12. Pery ARR, Mons R, Flammarion P, Lagadic L, Garric J (2002) A modelling approach to link food availability, growth, emergence and reproduction for the midge *Chironomus riparius*. Environmental Toxicology and Chemistry: 2507-2513.

13. Ali A, Berg MB, Coffman WP, Langton PH, Lindegaard C, et al. (1995) The Chironomidae: the biology and ecology of non-biting midges.; Armitage P, Cranston PS, Pinder LCV, editors. xii + 572 pp p.

14. Delettre Y, R. , Morvan N (2000) Dispersal of adult aquatic Chironomidae (Diptera) in agricultural landscapes. Freshwater Biology 44: 399-411.

15. Petersen I (2004) Dispersal of adult aquatic insects in catchments of differing land use. The Journal of applied ecology 41: 934.

16. Smith RF, Alexander LC, Lamp WO (2009) Dispersal by terrestrial stages of stream insects in urban watersheds: a synthesis of current knowledge. Journal of the North American Benthological Society 28: 1022-1037.

17. Oliver DR (1971) The life history of the Chironomidae. Annual Review of Entomology 16: 211-230.

18. Briggs CJ, Latto J (2000) The Effect of Dispersal on the Population Dynamics of a Gall-Forming Midge and Its Parasitoids. Journal of Animal Ecology 69: 96-105.

19. Barton KA, Phillips BL, Morales JM, Travis JMJ (2009) The evolution of an ‘intelligent’ dispersal strategy: biased, correlated random walks in patchy landscapes. Oikos 118: 309-319.

20. Hawkes C (2009) Linking movement behaviour, dispersal and population processes: is individual variation a key? Journal of Animal Ecology 78: 894-906.

21. Best DJ, Fisher NI (1979) Efficient Simulation of the von Mises Distribution. Journal of the Royal Statistical Society Series C (Applied Statistics) 28: 152-157.

22. Charles S, Ferreol M, Chaumot A, Pery ARR (2004) Food availability effect on population dynamics of the midge Chironomus riparius: a Leslie modeling approach. Ecological Modelling 175: 217-229.

23. Downe AER (1973) Some factors influencing insemination in laboratory swarms of *Chironomus riparius* (Diptera: Chironomidae). The Canadian Entomologist 105: 291-298.

24. Ducrot V, Pery ARR, Mons R, Garric J (2004) Energy-based modeling as a basis for the analysis of reproductive data with the midge (Chironomus riparius). Environmental Toxicology and Chemistry 23: 225-231.
