# Supplementary material for: Persistence of Aquatic Insects across Managed Landscapes: Effects of Landscape Permeability on Re-Colonization and Population Recovery
Source: PLoS One. 2013 Jan 24;8(1):e54584. doi: 10.1371/journal.pone.0054584 (PMC3554752; doi:10.1371/journal.pone.0054584)
Supplement: Supporting Information SII — Sensitivity analysis of the Chironomus riparius population model. (DOCX) [file pone.0054584.s006.docx]

**Supporting Information II**

**Sensitivity analysis of the *Chironomus riparius* population model**

We tested the output of the population model to changes in parameters. Here we focused on the impact temperature dependent growth and density-dependent mortality processes have on population abundance and recovery after stress.

As the result of different parameter sets, the abundance of control populations and population dynamics are presented. Daily abundances of the total population (from both simulated ditches) were averaged over the 3 years of simulation and presented in boxplots, with denoted median and interquartile ranges. Furthermore, we looked into how changes in the density-dependent mortality factors affect the time to recovery in treated populations. Populations in the treated ditch were exposed to extreme stress, i.e. 100% mortality, on day 150 (1^st^ June in the simulated year). Time to recovery was then estimated following the method described in the manuscript and below in the text.

From the dispersal model simulations, we obtained 3 main parameters (see the model ODD in SI I) that were used in the population model, namely the probability to stay in the natal ditch, the probability to colonize the new ditch and the probability to die in the landscape matrix between the ditches. Here, we keep the effects of dispersal constant and assume, for the purposes of this analysis, the dispersal mortality of 0, and equal probability to colonize a new ditch and stay in the native one.

This sensitivity analysis relates to the version 1.401 of the *Chironomus riparius* population model, implemented in the NetLogo platform [1].

1. **Temperature-dependent larval growth**

This species’ growth and development is highly dependent on available food and temperature of the surrounding environment. Its populations can be uni- to multivoltine, depending on local environmental conditions [2]. In this model, the population has 3 generations in a year, found also in Learner and Potter (1974) [3]. The temperature-dependent function is set up in such a way that the size increment exponentially increases with rising water temperatures, e.g. with the water temperature of 24° C, the increment added is maximal. This was also tuned to laboratory data on chironomid growth, where water temperatures are kept at a constant 21° or 25°; when the individuals are kept at 21°, one generation takes approximately 35 days [4], whereas it takes ca. 42 days in the population model (if the temperature is kept constant). Modelled individuals do not grow at all if the temperatures are lower than 8°. The water temperature values used in the model are based on measurements from the Dutch ditch system [2] and vary on a daily basis. The maximum value of the water temperature used in this model is 18.8°. We, therefore, assume yearly temperature dynamics, but do not account for possible interannual variability in temperatures, i.e. the same temperature set is used with each new simulation year.

Here we analyse the impact of increasing temperatures on voltinism (Figure SI II 1) and total population abundance (Figure SI II 2).

Increase in temperatures enhances the growth of larvae, resulting in shorter generation duration periods, when compared to the default parameter set (see SI I for details on the growth submodel). Ultimately, this yields more generations in a year (Figure SI II 1); note that only the third year of the simulation is shown. Here we plotted the default temperature set with the one where all daily temperatures were increased by 2° and by 6° C. With the temperature increase, new generations form; initially relatively small in size (due to few individuals reproducing), they grow as the temperatures increase even further.

Increase in the number of generations per year marginally increases the total population abundance (Figure SI II 2). The overall increase in abundance is minimal as the realized densities are mainly governed by the density-dependent mortality.

Increase in temperature causes faster larval growth and emergence and consequently, species in lower latitudes typically experience more generations in one year than species in higher latitudes ([5], and references therein). However, chironomids are also sensitive to microclimatic temperature conditions resulting in the same species exhibiting different number of generations in the same system [5].

1. **Density-dependent mortality**

In the population model, we include three different mortalities affecting individuals in the aquatic part of the life-cycle, background or natural mortality, density-dependent and pesticide-induced mortality. We assume daily mortality rates only for the larval stage (eggs and pupae have 100% survival).

Including density-dependent mortality is an indirect way of modelling resource competition, because we do not model resource dynamics. Even though exact mechanisms of density dependence are not clear, it is known that populations of chironomids are regulated by their densities, although these densities can be very high before any regulation occurs, e.g. Pery et al. (2002) [4] show that animals in beakers with higher numbers of individuals grow slower due to food limitation (beakers used had a surface are of 14 cm^2^  and the effects on growth were visible already with 10 individuals in one system). With ad libitum feeding conditions, density dependent effects seem to be much less pronounced. Hooper et al. (2003)[6] also show that at densities of 16 individuals/cm^2^, only 1% of individuals eventually emerges.

The realized densities in the model are lower than potentially attainable, due to computational reasons. The default value of 0.003 /ind per patch, results in the probability of dying of 1.0 if the density within the cell reaches 333 individuals. Consequently, the density of local populations (in a cell) is regulated.

Here we test the model output, i.e. total population abundance, while changing the strength of this parameter. Relaxing the impact of density-dependence results in higher population abundances (Figure SI II 3, dd_0.001). On the other hand, strengthening the density-dependent factor yields much lower abundances, and hampers populations’ survival over winter (Figure SI II 4, density-dependent mortality factor of 0.005 and 0.01). Population where this factor was highest (0.01) became extinct within the first simulation year.

Consequently, the default value was chosen such that the population abundances were computationally manageable, and that population densities were high enough so that they are not subject to demographic or environmental stochasticity.

Finally, we analysed the effects of different density-dependent factors on recovery times. We analysed only the following factors: 0.003 (default), 0.001 and 0.005.

The model output from populations exposed to stress was compared with that from control populations. For the analysis of recovery times after each of simulated exposures, we used 20 replicate simulations of treatment and control. Daily abundances of 20 treated populations were compared to 20 replicates of control populations, yielding potentially 400 recovery times. A treated population was considered to be recovered once its abundance reached or was higher than 95% of abundance of the control population; if this condition was met for five days within a ten day period, we deemed the population recovered. The day of recovery was then noted to be in the middle of this 10 day period.

Overall, recoveries were accomplished very fast in all cases (Figure SI II 4), with a median of 5 days. However, the upper ranges in boxplots imply that some populations (out of 20 replicates) took much longer to recover. When the consequence of the density-dependent factor was the increase in abundances, e.g. factor 0.001 (also Figure SI II 3), the upper limits to recovery times are the lowest, as effectively a higher population growth rate is allowed leading to faster recovery. Conversely, when lower abundances are realized, e.g. factor 0.005 (Figure SI II 3), some population may take much longer to recover.

References

1. Wilensky u (1999) NetLogo. Centre for Connected Learning and Computer-Based Modelling, Northwestern University, Evanston, Il.

2. Veraart AJ, de Klein JJM, Scheffer M (2011) Warming Can Boost Denitrification Disproportionately Due to Altered Oxygen Dynamics. PLoS ONE 6: e18508.

3. Learner MA, Potter DWB (1974) The seasonal periodicity of emergence of insects from two ponds in Hertfordshire, England, with special reference to the chironomidae (Diptera: Nematocera). Hydrobiologia 44: 495-510.

4. Pery ARR, Mons R, Flammarion P, Lagadic L, Garric J (2002) A modelling approach to link food availability, growth, emergence and reproduction for the midge *Chironomus riparius*. Environmental Toxicology and Chemistry: 2507-2513.

5. Eggermont H, Heiri O (2012) The chironomid-temperature relationship: expression in nature and palaeoenvironmental implications. Biological Reviews 87: 430-456.

6. Hooper HL, Sibly RM, Hutchinson TH, Maund SJ (2003) The influence of larval density, food availability and habitat longevity on the life history and population growth rate of the midge Chironomus riparius. Oikos 102: 515-524.
